# Relationship between lysosomal dyshomeostasis and progression of diabetic kidney disease

**DOI:** 10.1038/s41419-021-04271-w

**Published:** 2021-10-18

**Authors:** Man Wu, Minjie Zhang, Yaozhi Zhang, Zixian Li, Xingyu Li, Zejian Liu, Huafeng Liu, Xiaoyu Li

**Affiliations:** Institute of Nephrology, and Key Laboratory of Prevention and Management of Chronic kidney Disease of Zhanjiang City, Affiliated Hospital of Guangdong Medical University, Zhanjiang, Guangdong 524001 China

**Keywords:** Mechanisms of disease, Chronic kidney disease

## Abstract

Lysosomes are organelles involved in cell metabolism, waste degradation, and cellular material circulation. They play a key role in the maintenance of cellular physiological homeostasis. Compared with the lysosomal content of other organs, that of the kidney is abundant, and lysosomal abnormalities are associated with the occurrence and development of certain renal diseases. Lysosomal structure and function in intrinsic renal cells are impaired in diabetic kidney disease (DKD). Promoting lysosomal biosynthesis and/or restoring lysosomal function can repair damaged podocytes and proximal tubular epithelial cells, and delay the progression of DKD. Lysosomal homeostasis maintenance may be advantageous in alleviating DKD. Here, we systematically reviewed the latest advances in the relationship between lysosomal dyshomeostasis and progression of DKD based on recent literature to further elucidate the mechanism of renal injury in diabetes mellitus and to highlight the application potential of lysosomal homeostasis maintenance as a new prevention and treatment strategy for DKD. However, research on screening effective interventions for lysosomal dyshomeostasis is still in its infancy, and thus should be the focus of future research studies. The screening out of cell-specific lysosomal function regulation targets according to the different stages of DKD, so as to realize the controllable targeted regulation of cell lysosomal function during DKD, is the key to the successful clinical development of this therapeutic strategy.

## Facts

- AGE–RAGE interaction is a potential mechanism underlying lysosomal dysfunction.
- The screening out of cell-specific lysosomal function regulation targets according to different DKD stages is key to the successful clinical development of lysosomal homeostasis maintenance as a therapeutic strategy for DKD.
- Screening effective interventions for lysosomal dyshomeostasis should be the focus of future research efforts.

## Open Questions

- What’s the mechanisms of CTSD protecting PTECs from apoptosis and LMP?
- What’s the cell-specific lysosomal function regulation targets in different DKD stages?
- How to combine lysosome function regulation with cell-specific drug delivery?

## Introduction

Lysosomes are important regulatory platforms in numerous vesicle transport pathways, including endocytosis, phagocytosis, and autophagy. Their ability to fuse with endosomes, phagosomes, and autophagosomes enables them to break down a variety of endogenous and exogenous substances, including macromolecules, certain pathogens, and damaged organelles^1^. Owing to their central position in complex intracellular trafficking networks, lysosomes have become central signaling nodes for sensing and coordinating cellular metabolism, intra- and inter-cellular signaling, and membrane repair^1^.

Diabetic kidney disease (DKD) is a common consequence of type 1 diabetes mellitus (T1DM) and type 2 diabetes mellitus (T2DM)^2^. It has attracted much attention in recent years owing to its high incidence, poor prognosis, and heavy economic burden. DM-mediated changes in extracellular and intracellular metabolism and hemodynamics in intrinsic renal cells have a considerable and lasting impact on DKD progression^3^.

Autophagy protects intrinsic renal cells from injury and cell death in several renal diseases, including DKD^4–6^. Our previous study showed that lysosomal depletion induced by lysosomal membrane permeabilization (LMP) weakens intrinsic renal cell autophagy protection in DKD^7^. We found that long-term proteinuria (a secondary nephrotoxin factor that aggravates DKD progression) inhibited autophagy in intrinsic renal cells by impairing lysosomal structure and function^8^. Alternatively, preventing lysosomal depletion by eliminating damaged lysosomes or replenishing intact lysosomes improves autophagy flux, thereby protecting intrinsic renal cells from damage during DKD^9,10^.

The clinical application of lysosome dysfunction for the prevention and treatment target of DKD has not yet been realized. To promote the development of therapeutic lysosome targeting, in this review we summarize the latest advances in lysosomal dyshomeostasis in intrinsic renal cells, including podocytes, proximal tubular epithelial cells (PTECs), and macrophages during DKD, to highlight the key mechanism of interaction between lysosomal dyshomeostasis and progression of DKD.

## New understanding of lysosomal function and regulation

### Recent findings on lysosomal function

**Fig. 1:**
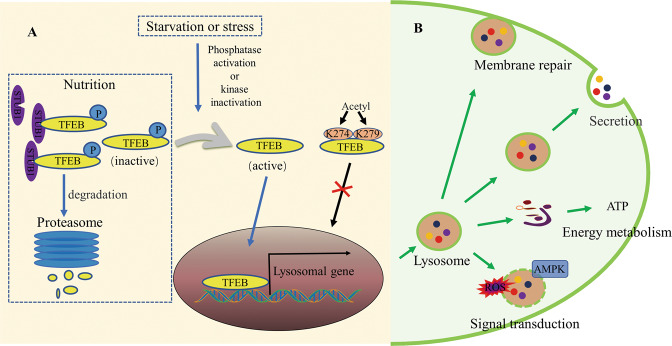
Schematic diagram of TFEB regulation and lysosomal function. **A** Under physiological (nutrient) conditions, phosphorylated TFEB is inactive and degradation occurs via the proteasomal pathway. Under starvation or stress, TFEB is activated by kinase inactivation or phosphatase activation-mediated dephosphorylation, and is subsequently transferred to the nucleus to upregulate the expression of its target genes. TFEB acetylation at K274 and K279 blocks TFEB nucleation^28–31,33^. **B** Lysosomes have several biological functions, including membrane repair, secretion, energy metabolism, and signal transduction^1,13–23^. AMPK, adenosine 5′-monophosphate (AMP)-activated protein kinase; ROS, reactive oxygen species; STUB1, STIP1-homologous U-Box containing protein 1; TFEB, transcription factor EB.

Lysosomes are important sub-organelles of single-layer capsulated vesicles containing >60 acidic hydrolases, including proteases, phosphatases, and lipases^11^. Lysosomes are involved in the degradation of cellular waste products, and play a crucial role in maintaining cellular homeostasis^12^. However, the role of lysosomal dysfunction in the pathogenesis of different diseases has not yet been elucidated, as lysosomes have only been considered end-degradation compartments involved in the elimination of cellular waste. Yet, lysosomes have several other important biological functions, including membrane repair, secretion, energy metabolism, and signal transduction (Fig. 1B)^1,13^.

An important marker of plasma membrane repair is the initiation of lysosome recruitment and exocytosis at the damaged site^14^. As lysosomes are able to respond to Ca^2+^ influx, rapid lysosomal exocytosis promotes plasma membrane repair, possibly by providing membrane support and tension release^14–16^. Lysosomes are essential for the maintenance of plasma membrane integrity, thus avoiding cytoplasm leakage and cell death.

Previously, lysosomal exocytosis was considered a unique cell secretory function, and myeloid cells were thought to contain a unique type of lysosome with exocytosis ability^17^. However, several subsequent studies showed that following the fusion of lysosomes with the plasma membrane, all cell types secrete lysosomal contents under different stimuli^18,19^.

Lysosomes are terminal degradation components that participate in the degradation of damaged cell structures, senescent organelles, and biological macromolecules, and produce small molecules (e.g., amino acids and fatty acids), which are subsequently transported to the cytoplasm for cell reuse and ATP generation to provide energy, thereby ensuring energy metabolism of cells^1,20^.

Lysosomal damage activates AMPK, a regulator of autophagy^21^. Upon lysosomal injury, galectin 9 increases the association with lysosomal glycoproteins, whereas it decreases interactions with the deubiquitinase USP9X; K63 ubiquitination of TAK1 activates AMPK in damaged lysosomes^22,23^. Furthermore, lysosomes also participate in immune processes^24^ and form exosomes^25^.

### Transcription factor EB (TFEB)-mediated lysosomal biogenesis regulation

Lysosomal genes share a 10-base E-box-like palindrome sequence (5′-GTCACGTGAC-3′), which is usually found within 200 bp of the transcription initiation site. This motif, termed coordinated lysosomal expression and regulation (CLEAR) element, consists of an E-box (CANNTG) identified by MIT/TFE family transcription factors (e.g., TFEB and TFE3)^26^. TFEB promotes transcription and expression of its target genes by specifically binding to the distinct CLEAR motif in the target promoter^26^, which increases the biogenesis of lysosomes and improves their degradation ability^27^.

TFEB activity primarily depends on its phosphorylation state and cytoplasmic-nuclear shuttling (Fig. 1A)^28^. Under physiological conditions, phosphorylated TFEB is inactive and predominantly located in the cytoplasm. Under stress, TFEB is activated by kinase inactivation or phosphatase activation-mediated dephosphorylation, and is subsequently transferred to the nucleus and combines with the CLEAR motif to upregulate its target gene expression^29,30^. TFEB degradation occurs via the proteasomal pathway^31^. Proteasome inhibition induces TFEB accumulation, dephosphorylation, and subsequent nuclear translocation, which remarkably increases the expression of TFEB downstream genes^32^. Wang et al. further reported that TFEB acetylation at K274 and K279 disrupted the dimerization of TFEB and its DNA‐binding activity, leading to inhibition of lysosome biogenesis^33^.

## DKD and lysosome function in intrinsic renal cells

### Epidemiological characteristics and primary pathogenesis of DKD

DKD is the leading cause of end-stage renal disease (ESRD). Up to 50% of patients with diabetes eventually develop DKD, leading to a considerable increase in the risk of mortality in these patients^34,35^. According to the International Diabetes Federation, the number of people with diabetes worldwide will increase from 382 million in 2013 to 592 million in 2035^36^, which will impose a huge economic burden on the family of the patients and on society.

DKD pathogenesis is extremely complex. Preliminary signs and symptoms of DKD generally appear 10–20 years after the onset of diabetes^37^. DKD occurs through various mechanisms, including hemodynamic changes, altered metabolic factors, and pro-inflammatory molecules^38^. Moreover, activation of the renal renin–angiotensin system (RAS)^39^, mitochondrial dysfunction^40,41^, and endoplasmic reticulum stress^40,42^ are also involved in DKD injury.

DKD pathogenesis has traditionally been characterized by glomerular pathology (e.g., mesangial hypertrophy, glomerular ultrafiltration, and proteinuria)^43^. However, recent research has suggested that glomerular alterations are not the main inducing factors of DKD, and renal tubular injury, especially proximal tubule injury, is likely to be a key inducer of major pathological events of progression to diabetic kidney failure^44^. The cellular morphology alteration of renal proximal tubules is considered an early symptom of DKD, and subsequent tubulo-interstitial fibrosis may play an important role in progression to end-stage renal failure^45,46^.

The prevention and treatment of DKD are conducted in a multi-target manner, including promoting a healthy lifestyle and targeting molecular factors associated with the pathogenesis of the disease. Intensive interventions (e.g., blood pressure control, glycemic control, and RAS suppression) reduce the risk of proteinuria progression; however, these therapies have failed to prevent DKD progression in some patients with refractory proteinuria^47^. Consequently, there is a necessity to find more effective treatment options to improve the prognosis of DKD.

### Emphasis of lysosomal dysfunction in DKD

The autophagy-lysosome pathway has been one of the main focuses of studies on DKD pathogenesis^48–52^. When renal cells are exposed to various stressors, such as oxidative stress, hypoxia, and toxic damage, autophagy is induced and plays an important role in cell survival^53–55^. However, reports on changes in autophagy activity in intrinsic renal cells during DKD are inconsistent. Zhan et al. showed that autophagy was inhibited in tubular epithelial cells (TECs) of diabetic animals^56^, whereas the opposite was observed by Zhao et al.^57^. Fang et al. suggested that hyperglycemia disrupted autophagy in podocytes^58^, whereas Ma et al. reported that high glucose (HG) promoted autophagy^59^. These differences may be caused because these studies primarily evaluated the upstream, rather than downstream, autophagy-lysosome pathway.

The autophagy-lysosome pathway involves autophagic vacuole induction, a fusion of autophagic vacuoles with lysosomes, and lysosomal degradation of autophagic vacuoles^60^. Lysosomes, located at the end of the autophagy-lysosome pathway, play an important role in the degradation of impaired organelles and macromolecules^61^. A decrease in lysosomal enzyme activity^62,63^ and lysosome-mediated degradation of albumin in the kidney^64^ under diabetic conditions have been reported. Furthermore, lysosomal damage leads to reduced renal protein degradation, resulting in diabetic renal hypertrophy^63^. Examination of renal parenchyma from 12 patients with DM showed a 50% reduction in TFEB mRNA levels versus 12 individuals without DM. A reduction in TFEB protein levels in the kidney tubulointerstitium was also observed in DM patients^65^. These findings suggest that considerable attention should be paid to the role of lysosomal dysfunction in DKD pathogenesis.

## Lysosomal dysfunction and enzyme abnormalities of podocytes in patients with DKD

Podocytes are highly differentiated cells on the lateral surface of the glomerular basement membrane and play an important role in maintaining the structure and function of the glomerular filtration barrier^66^. Podocyte dysfunction and loss because of apoptosis can contribute to massive proteinuria in DKD patients^67,68^. Various metabolites [e.g., advanced glycation end products (AGEs), uremic toxin, and methylglyoxal] can damage podocytes and lead to DKD^69–71^. Lysosomes participate in the processing of albumin in podocytes. Inhibition of lysosomal degradation can increase albumin accumulation, aggravating podocyte injury and glomerulosclerosis^72^. Consequently, lysosomal dysfunction in podocytes caused by etiological factors under diabetic conditions can aggravate podocyte lesions.

### Lysosomal degradation dysfunction

**Fig. 2:**
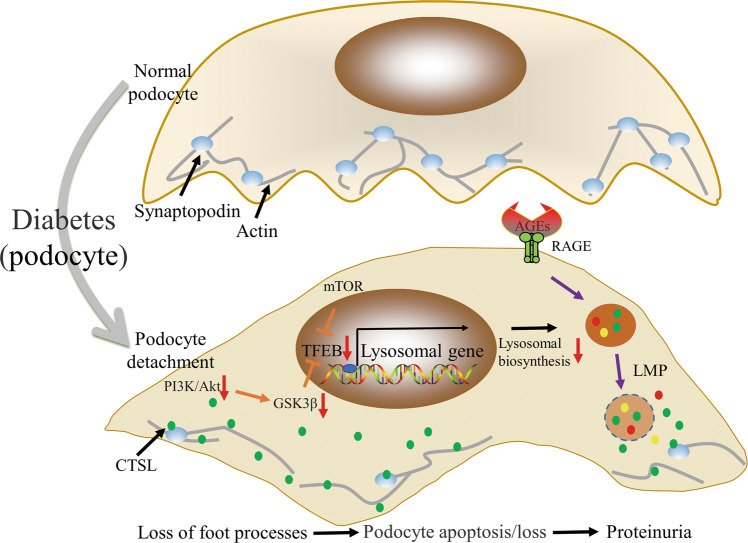
Mechanism of lysosomal dyshomeostasis and its effect on podocyte during DKD. LMP-related lysosomal degradation dysfunction is a crucial contributor to podocyte damage; mTOR inhibition and the PI3K/Akt-GSK3β axis prevent TFEB nuclear translocation and lysosomal biogenesis^73–76^. CTSL can degrade synaptopodin-inducing albuminuria by presenting a migration of podocyte foot processes^86–90^. AGEs, advanced glycation end products; CTSL, cathepsin L; DKD, diabetic kidney disease; LMP, lysosomal membrane permeabilization; RAGE, AGE receptor; TFEB, transcription factor EB.

Restoration of lysosomal function can activate podocyte autophagy and alleviate podocyte apoptosis in DKD^73^. Substantial time-dependent decreases in lysosomal enzyme activity [e.g., cathepsin B, D, and L (CTSB, CTSD, and CTSL)], LMP, and autophagy inhibition in podocytes were detected following AGE treatment. L-leucyl-L-leucine-O-methyl ester, known as an LMP inducer, inhibits autophagy in podocytes and aggravates podocyte apoptosis, revealing that LMP may be a critical factor triggering podocyte injury after AGE exposure under diabetic conditions. Notably, resveratrol plus vitamin E treatment in AGE-treated podocytes may increase CTSB/CTSL enzymatic activity and DQ-ovalbumin degradation, rescue actin cytoskeleton changes, and alleviate podocyte apoptosis. However, these cytoprotective effects were blocked by the addition of the lysosomal inhibitor leupeptin, suggesting that LMP-related lysosomal degradation dysfunction is a crucial contributor to podocyte damage during DKD occurrence and progression (Fig. 2)^73^.

### TFEB inactivation mediated lysosomal biogenesis obstruction

TFEB is the master regulator of the autophagy-lysosome pathway. Chen et al. proposed that catalpol stabilized the cytoskeleton, ameliorated podocyte injury, and recovered kidney damage in DKD by inhibiting mTOR activity and promoting TFEB nuclear translocation^74^. Phosphorylated p70s6k levels (p-p70s6k, a downstream target of mTOR) considerably increased in podocytes from DKD mice and cultured podocytes treated with HG content, with TFEB nuclear translocation also being affected. However, catalpol treatment reversed these changes. Zhao et al. demonstrated that AGEs triggered podocyte injury and pathological injury of the kidney by activating mTOR and subsequently inactivating TFEB^75^. Co-immunoprecipitation results indicated that TFEB interacted with mTOR in the glomeruli of db/db mice and AGE-stimulated podocytes. Torin1 (a strong inhibitor of mTOR activity) recovered TFEB nuclear expression in db/db mice and AGE-stimulated cultured podocytes. Hou et al. found that hepatocyte growth factor improved lysosome function by promoting TFEB nuclear translocation via the PI3K/Akt-GSK3β-TFEB axis in podocytes, which decreased urinary albumin excretion, alleviated matrix expansion, and rescued podocyte loss in DKD mice (Fig. 2)^76^. Collectively, TFEB activation, lysosomal biogenesis, and lysosomal function enhancement play key roles in preventing podocyte injury in DKD.

Inhibition of mTOR activity reduces podocyte injury by activating TFEB under diabetic conditions. However, inhibition of mTORC1 under non-diabetic conditions exerts serious adverse effects on podocytes^77,78^. The absence of mTORC1 activity in podocyte-specific Raptor-deficient mice resulted in severe podocyte injury, proteinuria, and glomerulosclerosis^79^. This highlights the importance of regulation of the mTOR signaling pathway to ensure normal renal function. The strong protective effect of activated TFEB on DKD should be emphasized, and other signaling pathways that activate TFEB should be actively explored.

### Abnormalities of lysosomal enzyme CTSL during DKD and its role in DKD pathogenesis

CTSL is a key lysosomal enzyme, and a cysteine protease of the cathepsin family^80^. Increased CTSL expression in podocytes was observed in patients with DKD, highlighting the clinical relevance of these findings^81^. Urine CTSL concentrations were lower in children with DKD than in children without DKD, which raises the possibility that CTSL may be an early predictor of DKD^82^. High CTSL levels in urine were associated with albuminuria improvement after four years of DKD diagnosis in patients^83^. The increase in serum CTSL activity positively correlated with the hospitalization rate of DKD patients, and serum CTSL levels positively correlated with proteinuria severity^84^. Regarding the role of CTSL in renal filtration function, studies have shown that CTSL inhibitors can reduce experimental proteinuria^85^.

A study using a streptozotocin (STZ)-induced CTSL-deficient diabetic mouse model and an STZ-induced wild-type (WT) diabetic mouse model showed that CTSL was associated with podocyte injury by aggravating proteinuria, mesangial matrix expansion, and tubular fibrosis^86^. Following diabetes induction, cortical CTSL activity and mRNA expression notably increased in WT mice^86^. CTSL-deficient diabetic mice did not develop albuminuria, displayed better renal function with normal plasma creatinine and blood urea nitrogen concentrations, and did not suffer from DKD^86^. Podocyte-specific calcineurin-CTSL interference was sufficient to induce albuminuria, also indicating that CTSL plays a key role in albuminuria pathogenesis^87^.

Proteinuria occurrence represented a migration of podocyte foot processes, caused by CTSL (Fig. 2)^88^. Notably, CTSL can degrade CD2-related proteins, synaptopodin, and dynamin, which are crucial for the normal architecture of the podocyte cytoskeleton^81,87,89^. Synaptopodin, an antagonist of RhoA and Cdc42 signaling, stabilizes renal filtration by preventing the podocyte actin cytoskeleton from reorganizing to form a migratory phenotype^90^. Consistently, synaptopodin protein expression was remarkably reduced in WT diabetic mice compared to CTSL-deficient diabetic mice^86^. Lysosomal CTSL plays an important role in DKD development by inducing albuminuria, which degrades important structural proteins in podocytes.

## Lysosomal dysfunction and enzyme abnormalities of PTECs in DKD

Phenotypic changes in renal PTECs are the first signs of DKD^91^, and the consequent tubulointerstitial injury plays a key role in the progression of DKD to ESRD^92,93^. AGEs are generated because of chronically high sugar levels, degraded by lysosomes via endocytosis in PTECs, and relevant to the progression of DKD^94^. In addition to elevated AGE production in the hyperglycemic state, the decreased lysosome clearance rate contributes to AGE accumulation in PTECs with DKD^95^.

### LMP occurrence

**Fig. 3:**
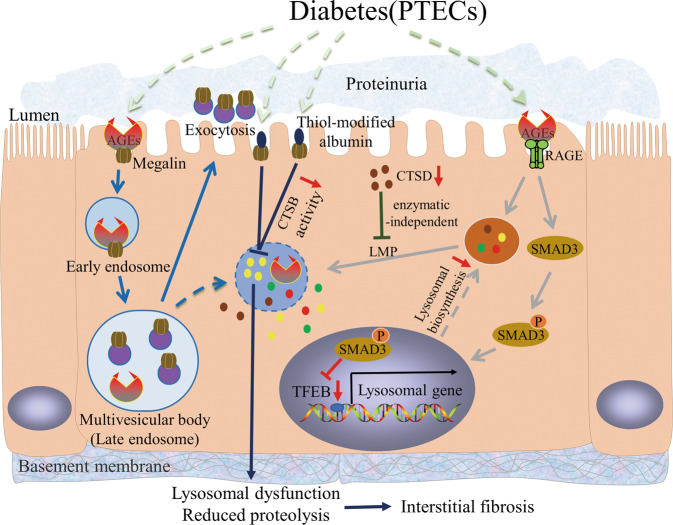
Mechanism of lysosomal dyshomeostasis and its effect on PTECs during DKD. AGEs can induce LMP through the AGE-RAGE interaction, triggering lysosomal dysfunction and tubular injury^7^. Smad3 binds with TFEB and inhibits its expression, resulting in inhibition of lysosome biosynthesis obstacle^95^. Megalin-mediated uptake of AGEs causes lysosomal dysfunction, leading to increased multivesicular body formation and exocytosis-mediated urinary megalin excretion. Renal PTECs lack protein endocytosis megalin receptors, resulting in proteinuria persistence^104,105^. LMP caused by AGEs can marginally improve by CTSD upregulation, independent of its enzymatic activity^112^. CTSB activity decreases by megalin-mediated uptake of thiol-modified albumin. Subsequently, the rate of lysosomal CTSB proteolysis is compromised, contributing to renal tubulointerstitial fibrosis^119^. AGEs, advanced glycation end products; CTSB, cathepsin B; CTSD, cathepsin D; DKD; diabetic kidney disease; LMP, lysosomal membrane permeabilization; PTECs, proximal tubular epithelial cells; RAGE, AGE receptor; TFEB, transcription factor EB.

Our previous study demonstrated the close association between lysosome impairment and abnormal AGE accumulation in PTECs and its subsequent contribution to tubular injury in DKD^7^. In human PTECs (HK-2), AGE exposure caused a significant reduction in CTSB and CTSL activity. The degradation ability of lysosomes was partially improved by anti-AGE receptor (RAGE) antibody pretreatment, demonstrating that the AGE–RAGE interaction is a potential mechanism underlying lysosomal dysfunction. AGE–RAGE axis played a crucial role in LMP occurrence by promoting oxidative stress generation^7^. Furthermore, quantitative cytoplasmic active cathepsins released from destructive lysosomes might play an important role in triggering TEC apoptosis and subsequent tubulointerstitial injury^96^. These results suggest that AGEs can induce LMP through the AGE–RAGE interaction, subsequently triggering further accumulation of AGEs and exacerbating DKD progression (Fig. 3).

Singh et al.^97^ investigated the urinary proteome of 220 adolescents and children; half had T1DM for an average of seven years and a half were healthy siblings. Eight of the fifteen proteins most substantially elevated in the T1DM cohort were lysosomal-related, including lysosomal enzymes or present in lysosomes, supporting the concept that damage of lysosomal membrane in PTECs occurs in T1DM patients. Urinary lysosomal enzyme loss and lysosomal enzymuria are early biomarkers of DKD^98^. Therefore, LMP or lysosomal dysfunction in PTECs must be considered during DKD development.

### Lysosomal dysfunction results in increased urinary protein excretion

Proteinuria is the result of increased glomerular capillary wall permeability and/or decreased proximal tubule endocytosis^99,100^. Under normal physiological conditions, the proximal tubule plays an important role in reabsorbing filtered low-molecular-weight (LMW) proteins (<65 kDa) and albumin via an accurate mechanism of receptor-mediated endocytosis (e.g., megalin and cubicin, two types of multiligand receptors)^101^. The dysfunction of renal proximal tubular endocytosis in DKD animals is associated with the excretion of high levels of urinary total protein, albumin, and transferrin^102^.

Megalin is involved in AGE uptake filtered by glomeruli in PTECs^103^. In a high-fat diet (HFD) diabetic mouse model, megalin-mediated lysosomal dysfunction in PTECs by ingestion of pathogenic ligands led to dysfunctional renal tubules compared with the megalin-KO mice group^104^. In AGE-treated cultured immortalized rat proximal tubule cells (IRPTCs), megalin-mediated uptake of AGEs caused lysosomal dysfunction, leading to increased multivesicular body (MVB) formation and exocytosis-mediated urinary megalin excretion (Fig. 3)^105^. Exosomes are formed in the endosomal network, with late endosomes fusing with lysosomes for further degradation or with plasma membranes for exosome excretion. Lysosomal dysfunction leads to increased MVB production, megalin recruitment in MVB, and release of exosomes containing megalin^105^. When IRPTCs were treated with two potent lysosome inhibitors, bafilomycin A1 (BAFA) and chloroquine (CQ) phosphate, lysosomal dysfunction increased MVB formation and exocytosis-mediated urinary megalin excretion^105^. Figueira et al. showed reduced mRNA and protein levels of the endocytic apparatus (e.g., megalin and cubicin) in the renal cortex and proximal tubules in the early stages of T1DM^102^. Urinary full-length megalin excretion via extracellular vesicles increased in HFD-fed mice^105^. The urinary level of megalin positively correlated with the DKD progression in T2DM patients^105^.

A urinary megalin ELISA has potential value for early diagnosis and severity assessment of DKD in patients with T2DM^106^. Notably, lysosomal dysfunction can be caused by megalin-mediated uptake of toxic ligands in DM, and increased megalin excretion in urine is because of lysosomal dysfunction in proximal tubules. Renal PTECs lack protein endocytosis receptors, resulting in proteinuria persistence, which accelerates DKD progression^107^. Therefore, these studies highlight the importance of lysosome function in proximal tubules to maintain endocytic protein receptor integrity, and the key role of lysosomes in DKD pathophysiology and diagnosis.

### TFEB-induced lysosomal biosynthesis alleviates autophagy stress in PTECs

Takahashi et al. demonstrated that more AGEs accumulated in the tubular cells of diabetic STZ-treated Atg5-deficient mice compared to control mice^6^. The protein levels of Lamp1 and TFEB, and nuclear translocation of TFEB, were decreased in AGE-exposed Atg5-deficient PTECs and tubular cells of diabetic Atg5-deficient mice. This indicated that autophagy contributed to the upregulation of TFEB expression, while promoting nuclear translocation of TFEB and lysosomal biogenesis to clearing the accumulated AGEs, which also highlights the importance of TFEB expression and lysosomal biogenesis in DKD.

We found that lysosomes in PTECs occur via LMP in the diabetic state^7,8^. Severely damaged lysosomes can be removed by lysophagy, and normal lysophagy propulsion requires intact and functional lysosomes^108,109^. Unfortunately, our in vivo and in vitro studies found that the number and proportion of primary lysosomes decreased with lysosome accumulation in diabetic PTECs. We found that Smad3 inhibited TFEB expression in DKD (Fig. 3), whereas inhibition of SMAD3 activity increased TFEB expression and rescued PTEC lysosomal renewal disorder in DKD^95^. Zhao et al. demonstrated that high-dose vitamin E therapy could alleviate autophagic stress, ameliorate proteinuria, and improve renal function in diabetic rats^110^. Autophagic stress is a continuous imbalance in which the formation rate of autophagosomes exceeds its lysosomal pathway degradation rate^111^. Interestingly, vitamin E treatment reduced the accumulation of autophagic vacuoles and autophagic substrates. Notably, the decline in CTSB and CTSL activity caused by AGE exposure was reversed following vitamin E treatment^110^. Thus, the improvement of lysosomal biosynthesis and lysosomal function provides a promising new option for DKD treatment to alleviate autophagic stress.

### CTSD improves PTEC injury independent of enzymatic activity

CTSD is a major aspartate protease within lysosomes^80^. Du et al. demonstrated that CTSD protected renal PTECs from injury induced by HG and AGE exposure, independent of its enzymatic catalytic activity under diabetic conditions^112^. Immunohistochemical staining of renal tissues from DM patients and non-DM subjects revealed relatively low expression of CTSD with a uniformly distributed pattern in non-DM tubules, whereas this was considerably reduced in disordered DM tubules. CTSD overexpression showed elevated cell viability and decreased apoptosis following AGE exposure in CTSD-transduced HK-2 cells. Furthermore, when HK-2 cells were treated with pepstatin A, a CTSD inhibitor, or transduced with a lentiviral vector encoding an inactive mutant of CTSD, the protective effect of CTSD persisted, indicating that CTSD protection is independent of its enzymatic activity^112^.

LMP contributes to CTSD release from lysosomes into the cytoplasm, and consequently triggers the mitochondrial apoptotic cascade^113^. Here, LMP caused by AGEs in HK-2 cells improved marginally by CTSD upregulation, indicating a potential benefit of CTSD in tubular damage in DKD (Fig. 3)^112^. Thus, the mechanisms by which CTSD protects PTECs from apoptosis and LMP require further investigation.

### Impaired CTSB activity inhibits protein degradation

CTSB is the primary lysosomal cysteine protease involved in lysosomal protein degradation and is primarily expressed in the cytoplasmic vesicles of PTECs in renal tissues^114^. CTSB promotes the degradation of reabsorbed urine protein, which is internalized by proximal tubular endocytosis. The reabsorbed urine protein is degraded in lysosomes to LMW fragments and returned to the tubular lumen^115^. A decrease in CTSB activity in PTECs accompanied by excretion of high-molecular-weight urinary proteins was noted in early DKD rats^116^. The degradation rate of long-lived proteins in PTECs was drastically reduced, accompanied by decreased CTSB activity, which led to the inhibition of protein degradation in PTECs, ultimately leading to diabetic renal hypertrophy^63^. CTSB activity considerably decreased when exposed to AGEs, resulting in decreased cell viability of PTECs^114^.

AGEs can inhibit protein degradation by inhibiting CTSB expression and activity^117^. In the STZ-induced DKD rat model, Sebekova et al.^118^ found that the cell volume of PTECs incubated with AGEs increased remarkably, intracellular and extracellular protein synthesis increased, protein degradation rate decreased, and CTSB activity decreased. A correlation between AGEs and CTSB was established by LMP occurrence when exposed to AGEs, resulting in lysosomal alkalization and CTSB inactivation^7^. Furthermore, Medina-Navarro et al. demonstrated that reduced CTSB activity in PTECs with DKD was closely related to the tertiary structure of reabsorbed albumin (Fig. 3)^119^. Albumin collected from stage 4 DKD patients presented >50% higher thiol-dependent changes in the albumin tertiary structure than that collected from stages 0 and 1. CTSB activity in isolated lysosomes revealed a significant inhibitory effect in HK-2 cells treated with albumin from stage 4 DKD patients and with albumin that was intentionally modified^119^. Subsequently, the rate of lysosomal CTSB proteolysis was compromised; thus, an imminent overload of proteins was induced, which facilitated transdifferentiation of epithelial tubular cells to myofibroblasts and contributed to renal tubulointerstitial fibrosis and DKD progression^119^. These findings suggest that CTSB may function as a therapeutic target in DKD.

## Macrophage iysosomal dysfunction and enzyme abnormalities in DKD

DKD is a chronic inflammatory disease characterized by numerous inflammatory cell infiltrates and overexpression of pro-inflammatory factors^120,121^. Numerous clinical and animal studies have found that most patients or animal renal tissues with DKD are associated with different levels of macrophage infiltration^122,123^. The degree of macrophage infiltration is associated with renal damage severity^123,124^. Therefore, the prevention of macrophage infiltration is crucial for DKD treatment.

### Normal iysosomal function alleviates macrophage infiltration and renal injuries

**Fig. 4:**
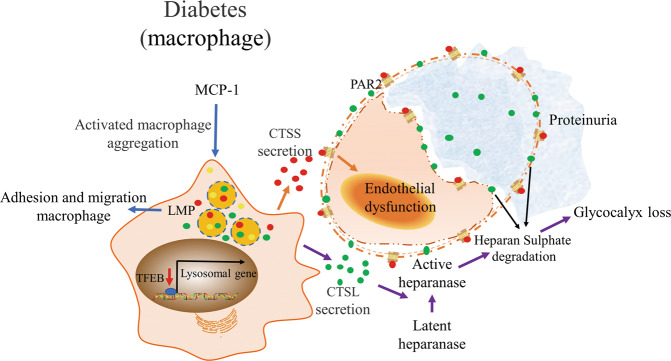
Mechanism of lysosomal dyshomeostasis in macrophages and its effect on renal endothelial cells during DKD. Lysosomal dysfunction promotes adhesion and migration of macrophages^125^. Decreased TFEB activity in macrophages is involved in kidney injury^126^. MCP-1 promotes activated macrophage glomerular aggregation^133,134^. Macrophage-derived CTSS could induce diabetic renal endothelial cell damage by activating PAR2 on the surface of endothelial cells^132^. CTSL can be secreted into the extracellular space of the kidney by glomerular macrophage infiltration, resulting in renal heparanase activation and subsequent glycocalyx loss and proteinuria^137,138^. CTSL, cathepsin L; CTSS; cathepsin S; DKD, diabetic kidney disease; MCP-1, monocyte chemotactic protein-1; PAR2, protease-activated receptor-2; TFEB, transcription factor EB.

Normal lysosomal function plays a crucial role in maintaining and controlling adhesion and migration of macrophages, thus alleviating renal injury. In a diabetic rat model, macrophage infiltration increased in renal tissue, characterized by the macrophage marker CD68^+^. In an in vitro experiment, adhesion and migration of macrophages increased under HG stimulation. The number of adhesion and migration macrophages further increased when BAFA or CQ were added simultaneously, compared with the HG group^125^. Furthermore, Yuan et al. reported that mesenchymal stem cells could elicit M1/M2 macrophages into the M2 phenotype and alleviate renal injury in DKD mice by activating TFEB and subsequently restoring the lysosomal substrate degradation function in macrophages (Fig. 4)^126^.

### Macrophage-derived CTSS induces endothelial cell damage

CTSS is a member of the cysteine cathepsin protease family. CTSS degrades proteins via the endosomal/lysosomal pathway and can be secreted into the extracellular environment for biological activity^127^. Unlike that of the other members of the cathepsin family, the enzyme activity of CTSS operates within a large pH range such that it can function outside the cell^128^. A prominent feature of CTSS is that its expression is primarily limited to leukocyte subsets, especially macrophages^129^. Elevated serum CTSS levels increase the risk of T2DM, suggesting a clinical correlation between CTSS suppression and reducing or delaying type 2 diabetes and kidney disease development in obese individuals^130^. In patients with ESRD, followed by an increase in the serum level of CTSS, the glomerular filtration rate and cysteine protease inhibitor C level decreased, indicating that CTSS activity increased with the progression of chronic kidney disease^131^.

Kumar et al. demonstrated that macrophage-derived CTSS could induce diabetic renal endothelial cell damage by activating protease-activated receptor-2 on the surface of endothelial cells (Fig. 4)^132^. In db/db mice, proteinuria, glomerulosclerosis, and renal inflammation were alleviated by treatment with RO5461111, a selective CTSS activity inhibitor, and PAR2 inhibition was effective in attenuating glomerulosclerosis. In an in vitro experiment, CTSS specifically triggered epithelial cell dysfunction through PAR2; however, it had no effect on TECs and podocytes^132^. Collectively, the inhibition of CTSS-mediated PAR2 activation in endothelial cells plays an important role in the prevention and improvement of DKD.

### MCP-1/Macrophage-derived CTSL injures glomerular barrier

Monocyte chemotactic protein-1 (MCP-1) is associated with monocyte recruitment from the circulatory system and monocyte and macrophage migration^133,134^. In patients with DKD, increased MCP-1 levels in renal tissue and urine suggested that macrophages play an important role in DKD development^135^. Glomerular macrophages have been observed in biopsies of mild DKD, suggesting that macrophages play a role in early diabetes-induced impairment^136^.

Boels et al.^137^ demonstrated that CTSL could be secreted into the extracellular space of the kidney by glomerular macrophage infiltration, making them candidates for renal heparanase activation and subsequent glycocalyx loss (Fig. 4). MCP-1 inhibition can reduce albuminuria and restore glomerular endothelial glycocalyx in DKD. Glomerular heparanase and CTSL protein expression was increased in diabetic mice, whereas it was decreased in diabetic mice treated with NOX-E36 (emapticap pegol, an MCP-1 inhibitor)^137^. Previous immunohistochemical studies showed that the macrophage marker F4/80 co-localizes with CTSL^138^. The above studies suggest that MCP-1 inhibition can restore glomerular barrier function by influencing macrophage CTSL secretion and reducing heparanase activation, highlighting the potential pathogenicity of CTSL released from infiltrated renal macrophages in DKD.

## Conclusions and future prospects

Despite the use of multiple intensive therapies, the incidence of diabetes continues to increase globally. DKD, a serious diabetic complication, is still the primary cause of ESRD, with high morbidity and mortality. Therefore, there is an urgent need to identify new effective therapeutic targets for the prevention and treatment of DKD. In this review, we provide an in-depth understanding of the role of lysosomes in DKD pathogenesis, identify potential mechanisms that promote the regulation of lysosome biogenesis and function in diabetic kidneys, and highlight a new research direction for the effective control of diabetic nephropathy.

However, valuable results of research on pathogenesis should eventually be applied to improve the treatment and prognosis of diseases. Currently, research on screening effective interventions for lysosomal dyshomeostasis is still in its infancy, and thus should be the focus of future research studies. The screening out of cell-specific lysosomal function regulation targets according to the different stages of DKD, so as to realize the controllable targeted regulation of cell lysosomal function during DKD, is the key to the successful clinical development of this therapeutic strategy.

## Data Availability

Data sharing is not applicable to this article as no datasets were generated or analyzed during the current study.
